# Visual Servoing of a Moving Target by an Unmanned Aerial Vehicle

**DOI:** 10.3390/s21175708

**Published:** 2021-08-25

**Authors:** Ching-Wen Chen, Hsin-Ai Hung, Po-Hung Yang, Teng-Hu Cheng

**Affiliations:** Department of Mechanical Engineering, National Yang Ming Chiao Tung University, Hsinchu 30010, Taiwan; amyking90511@gmail.com (C.-W.C.); louise.me08g@nctu.edu.tw (H.-A.H.); s933611@hotmail.com (P.-H.Y.)

**Keywords:** unscented kalman filter, quadratic programming, estimation, tracking of moving targets, UAV

## Abstract

To track moving targets undergoing unknown translational and rotational motions, a tracking controller is developed for unmanned aerial vehicles (UAVs). The main challenges are to control both the relative position and orientation between the target and the UAV to within desired values, and to guarantee that the generated control input to the UAV is feasible (i.e., below its motion capability). Moreover, the UAV is controlled to ensure that the target always remains within the field of view of the onboard camera. These control objectives were achieved by developing a nonlinear-model predictive controller, in which the future motion of the target is predicted by quadratic programming (QP). Since constraints of the feature vector and the control input are considered when solving the optimal control problem, the control inputs can be bounded and the target can remain inside the image. Three simulations were performed to compare the efficacy and performance of the developed controller with a traditional image-based visual servoing controller.

## 1. Introduction

Unmanned aerial vehicles (UAVs) have attracted much attention since the agileness made them capable of adapting to diverse terrains and executing various tasks such as monitoring, rescue, and target tracking [1,2]. Studies of UAVs have focused on localizing a target from sensing data of cameras [3], radars [4], and sensor networks [5,6,7]. Due to the advantageous availability and cost of cameras, many approaches can reconstruct the environment by 2D image features. Algorithms for estimating the position of objects based on image features captured by stationary cameras [8] and moving cameras [9] have been presented. Methods have been developed to reconstruct the 3D model of an object from a perspective camera [10,11]. Other features such as lines [12], cylinders and spheres [13], or planes [14,15] are also applied to a structure-from-motion (SfM) task. However, the aforementioned SfM methods rely on triangulation to recover the depth, and extension of these works to the motion estimation of moving targets is still challenging.

Stationary targets: Image-based visual servoing (IBVS) is a system control method that guarantees a series of visual features of a target will converge to the desired setpoints in the image [16]. However, IBVS approaches have potential problems such as causing larger tracking errors or losing the tracking altogether, especially when the target motion is time-varying or not predicted correctly [17]. Visual predictive control (VPC) [18,19] is a method that combines model predictive control constraints such as in the field of view (FOV), output limitations of the actuator, and workspace. A nonlinear model predictive controller was applied to an underwater vehicle to generate a desired velocity while satisfying the constraints of the visibility [20]. Similarly, MPC has been used to control a mobile robot while tracking a stationary feature point [21], to keep a visual feature of the target in the curtain position of the image [22,23], and to maximize the visibility of a target and minimize the velocity of its image feature when utilizing quadrotors. Artificial patterns have been used to predict all of the missing feature points due to occlusion problems and to ensure the execution of IBVS for navigation [24]. The aforementioned MPC approaches all considered only stationary targets, and so the present work designed an MPC to track a moving target, and moreover incorporated its motion estimation into the control sequence as a feedforward term in order to generate smooth control inputs.

Dynamic targets: Knowledge of motion of targets is crucial in tracking control; however, the knowledge is unknown and difficult to predict, and so various forms of image features are used for feedback control as to keep the target in FOV [25,26]. Many image processing methods have been developed for feature extraction and matching, such as RGB-based methods [27,28], scale-invariant feature-transform (SIFT) [29,30], and speeded-up robust-features (SURF) [31,32]. However, there are some drawbacks for these methods in terms of object detection and estimation of the relative motion between the camera and the target. Visual-based optimization methods have been applied a quadrotor [33], and it aimed to track a moving target while avoiding obstacles and the target position is assumed to be known in advance. Model-based optimization was applied to achieve perception and action objectives for robust sensing of a UAV [22]; however, the image features were extracted for indoor localization, rather than target tracking. Bounding boxes and feature points have been utilized as image features for tracking human beings [34,35,36,37], but the targets were moving at speeds that were so low that their motions could be ignored during tracking. Moreover, the orientation of the target is unavailable [38]. MPC has been applied to track a target moving along a periodic path, with this motion constraint reducing the complexity of the controller design [39]. However, the aforementioned works did not take the interaction between the target and the UAV into account, and the relative angle between the camera and the target was not modeled or measured.

This work models the interaction between a UAV and target and includes it in the developed controller, so that the tracking performance can be improved given certain motion, control, and FOV constraints. A DNN (e.g., YOLO [40]) is designed and trained to generate a bounding box in the image that encloses the target and predicts the angle of the target as viewed by the UAV, since both types of information are required to estimate target motion in an unscented Kalman filter (UKF). Finally, a high-order polynomial that emulates the target motion is obtained using quadratic programming (QP), which is used in the controller to predict the future motion of the target. The contributions of this work can be summarized as follows:

1. An optimal tracking controller is developed to track a moving target undergoing unknown motion while meeting motion, control, and sensing constraints, where the relative motion between the target and the UAV is estimated.
2. In contrast to previous approaches [34,35,36,37,38], this work models the dynamics of the relative rotation between the target and the UAV, and the relative angle can be controlled to a predefined desired value, which can be applied in applications such as the automatic searching, detection, and recognition of car license plate.
3. The controller is designed to ensure that the target remains within the FOV.
4. Compared to the IBVS controller, the developed controller can ensure smooth control input, less energy, and smaller tracking error.
5. The developed control architecture can be applied to track other moving targets as long as it can be detected by the YOLO network.

The organization of this work is as follows: Section 2 formulates the interaction between the target and the UAV as well as the control objectives. Section 3 describes how the target velocity is estimated based on the UKF and the bounding box in the image. Section 4 designs a controller to meet the control objectives. Simulations are presented in Section 5 to verify the efficacy of the developed controllers.

## 2. Preliminaries

### Control Objectives

**Figure 1:**
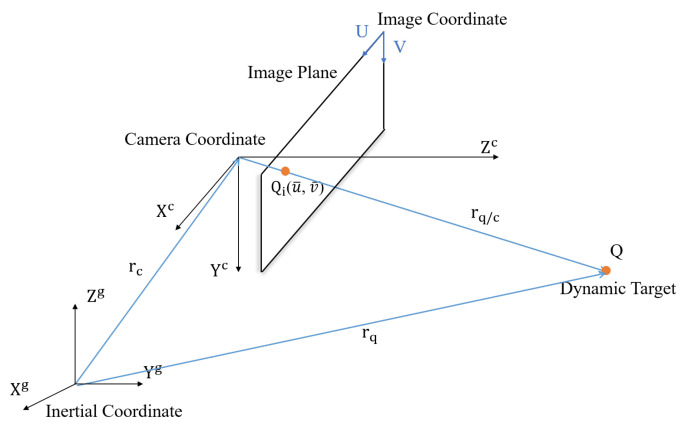
Kinematics model.

Figure 1 depicts the kinematics of a dynamic target and a camera fixed on a UAV, and the superscript G and the subscript *C* denote the inertial frame and the camera frame, respectively. The vector between the target and the camera can be expressed as

(1) $$\begin{array}{cc} r_{q/c} & =r_{q}-r_{c}, \end{array}$$

where $r_{q}={\left[\begin{array}{ccc} x_{q}, & y_{q}, & z_{q} \end{array}\right]}^{T}$ is defined as the target position, $r_{c}={\left[\begin{array}{ccc} x_{c} & y_{c} & z_{c} \end{array}\right]}^{T}$ is the camera position, $r_{q/c}=\left[\begin{array}{ccc} X, & Y, & Z \end{array}\right]$ is defined in the camera frame in order to facilitate the subsequent analysis, and *Z* defined in $r_{q/c}$ is the depth d∈R. Based on (1), the relative velocity ${\dot{r}}_{q/c}$ can be expressed as

(2) $$\begin{array}{ccc} {\dot{r}}_{q/c} & = & V_{q}-V_{c}-\omega_{c}\times r_{q/c}, \end{array}$$

where $V_{c}≜{\left[\begin{array}{ccc} v_{cx} & v_{cy} & v_{cz} \end{array}\right]}^{T}$ and $\omega_{c}≜{\left[\begin{array}{ccc} \omega_{cx} & \omega_{cy} & \omega_{cz} \end{array}\right]}^{T}$ are defined as the translational and angular velocities of the camera. In (2), $V_{q}={\left[\begin{array}{ccc} v_{qx} & v_{qy} & v_{qz} \end{array}\right]}^{T}$ denoted the translational velocity of the target is unknown and will be estimated, and $V_{c}$ and $\omega_{c}$ are the control input to be designed in the subsequent sections.

**Figure 2:**
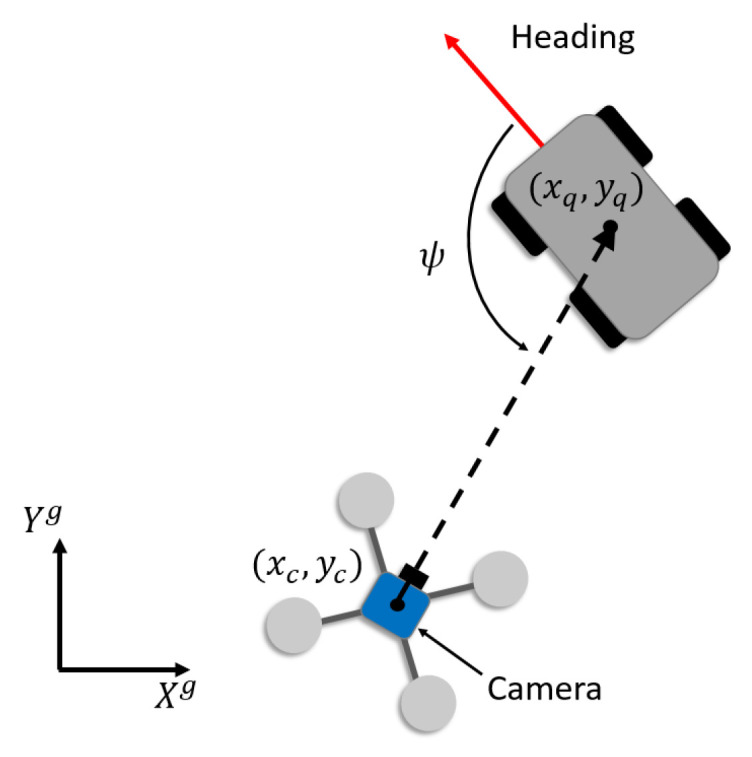
The definition of the orientation of target ψ observed by the UAV.

The orientation of the target denoted as ψ∈R shown in Figure 2 can be expressed as:

(3) $$\psi =\arctan\left(\frac{y_{c}^{g}-y_{q}^{g}}{x_{c}^{g}-x_{q}^{g}}\right)-\arctan\left(\frac{V_{qy}^{g}}{V_{qx}^{g}}\right),$$

where the superscript denotes the value defined in the global frame. Taking the time derivative on both sides of (3) yields the relative angular velocity between the target and the UAV:

(4) $$\begin{array}{cc} \dot{\psi }= & \frac{\left(v_{cy}^{g}-v_{qy}^{g}\right)\left(x_{c}^{g}-x_{q}^{g}\right)-\left(v_{cx}^{g}-v_{qx}^{g}\right)\left(y_{c}^{g}-y_{q}^{g}\right)}{{\left(x_{c}^{g}-x_{q}^{g}\right)}^{2}+{\left(y_{c}^{g}-y_{q}^{g}\right)}^{2}}\\ \; & -\frac{a_{qy}^{g}v_{qx}^{g}-a_{qx}^{g}v_{qy}^{g}}{{\left(v_{qx}^{g}\right)}^{2}+{\left(v_{qy}^{g}\right)}^{2}}. \end{array}$$

The control objective is to achieve

(5) $$r_{q/c}\to {\left[0,\;0,\;d_{des}\right]}^{T},\;\psi \to \psi_{des},$$

where implies that the target remains at the center of the image, and $d_{des}\;\mathrm{and}\;\psi_{des}\in R$ are the desired depth and angle, with respect to the target. The limitation of existing results is relaxed by making Assumption 1.

**Assumption** **1.** *$r_{q},{\dot{r}}_{q},{\ddot{r}}_{q}\in L_{\infty }$.*

## 3. Estimating the Motion of the Moving Target

### 3.1. Kinematics Model

To model the tightly coupled motion between the camera and the target, the states of the system are defined as

(6) $$x={\left[x_{1},\;x_{2},\;x_{3},\;\psi ,\;r_{q}^{T},\;V_{q}^{T}\right]}^{T},$$

where

(7) $$\left[x_{1},\;x_{2},\;x_{3}\right]=\left[\frac{X}{Z},\;\frac{Y}{Z},\;\frac{1}{Z}\right]$$

denotes the state related to the image feature. By taking the time derivative of (6), the dynamics of the visual servoing system can be obtained as

(8) $$\begin{array}{cc} \dot{x} & =\left[\begin{array}{c} v_{qx}x_{3}-v_{qz}x_{1}x_{3}+\zeta_{1}+\eta_{1}\\ v_{qy}x_{3}-v_{qz}x_{2}x_{3}+\zeta_{2}+\eta_{2}\\ -v_{qz}x_{3}^{2}+v_{cz}x_{3}^{2}-\left(\omega_{cy}x_{1}-\omega_{cx}x_{2}\right)x_{3}\\ \frac{\left(v_{cy}^{g}-v_{qy}^{g}\right)\left(x_{c}^{g}-x_{q}^{g}\right)-\left(v_{cx}^{g}-v_{qx}^{g}\right)\left(y_{c}^{g}-y_{q}^{g}\right)}{{\left(x_{c}^{g}-x_{q}^{g}\right)}^{2}+{\left(y_{c}^{g}-y_{q}^{g}\right)}^{2}}-\frac{a_{qy}^{g}v_{qx}^{g}-a_{qx}^{g}v_{qy}^{g}}{{\left(v_{qx}^{g}\right)}^{2}+{\left(v_{qy}^{g}\right)}^{2}}\\ V_{q}\\ \begin{array}{c} 0_{3\times 1} \end{array} \end{array}\right], \end{array}$$

where (2) is used and $\zeta_{1}$, $\zeta_{2}$, $\eta_{1}$, $\eta_{2},x_{c/q},y_{c/q}\in R$ are defined as

$$\begin{array}{cc} \zeta_{1} & =\omega_{cz}x_{2}-\omega_{cy}-\omega_{cy}x_{1}^{2}+\omega_{cx}x_{1}x_{2}\\ \zeta_{2} & =-\omega_{cz}x_{1}+\omega_{cx}+\omega_{cx}x_{2}^{2}-\omega_{cy}x_{1}x_{2}\\ \eta_{1} & =\left(v_{cz}x_{1}-v_{cx}\right)x_{3}\\ \eta_{2} & =\left(v_{cz}x_{2}-v_{cy}\right)x_{3}\\ x_{c/q} & =x_{c}-x_{q}\\ y_{c/q} & =y_{c}-y_{q}. \end{array}$$

### 3.2. Measurements: Bounding Box and Orientation

**Figure 3:**
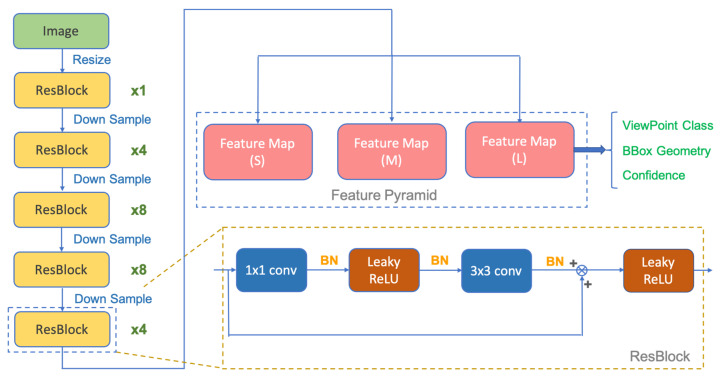
The network structure of a YOLO network, which take resized images as inputs and can generate three outputs, i.e., pose, bounding box, detection.

This section defines two measurements performed using a YOLO DNN as depicted in Figure 3. The bounding box and the orientation measurement provide information to correct the states in the UKF, and the detailed description of the DNN can be found in [41].

However, it is hard to defined the accuracy of the estimation since the output of the DNN network is classified into 24 classes. For example, as shown in the video (https://www.youtube.com/watch?v=KMQD7KzsnPE&list=PLrxYXaxBXgRqAUZyX5TsTsPaBtfSYFpHL&index=15) (accessed on 5 June 2021), the upper window demonstrates the estimation of the angle of the target, which is classified into 24 classes from 0 to 360 degree in order to reduce computational efforts. Therefore, when the camera moved from the left to the front, the angle only experienced 6 classes, and the accuracy calculated by comparing the differences between the 6 classes and the continuous variation of angles is not rigorously defined in the existing literature. Nevertheless, the estimation is affordable to be used for sensing the angle of the target in our application.

#### 3.2.1. Bounding Box

Based on the pinhole model, states $x_{1}$ and $x_{2}$ can be obtained as

$$\begin{array}{cc} x_{1} & =\frac{\bar{u}-c_{u}}{f_{x}} \end{array}$$

(9) $$\begin{array}{cc} x_{2} & =\frac{\bar{v}-c_{v}}{f_{y}} \end{array}$$

(10) $$\begin{array}{cc} x_{3} & =\frac{1}{d}, \end{array}$$

where $f_{x}$ and $f_{y}$ are the focal lengths of the camera, $\left[c_{u},\;c_{v}\right]$ represents the center of the image frame, $\bar{u}$ and $\bar{v}$ represent the center of bounding box that encloses the detected moving target in the image frame as depicted in Figure 1, and *d* is the depth obtained from the pinhole model

(11) $$\begin{array}{ccc} d & = & \sqrt{\frac{Af_{x}f_{y}}{a}}, \end{array}$$

where *a* is the area of the bounding box, and *A* is the ground truth side area of the target (i.e., typical size for a sedan is 4.6 m × 1.5 m).

**Remark** **1.** *The estimate of distance to the vehicle is obtained by (11) and is not directly generated from the DNN. That is, given the generated bounding box, its area A (i.e., unit in pixel) can be calculated and used to calculate the depth by (11). Therefore, as long as the generated bounding box can accurately enclose the target in the image and and the focal lengths are accurately obtained by calibration, the estimated depth will be accurate.*

Based on the camera measurement, pinhole model, orientation of the target, and the location of the UAV, the measurement model can be derived as

(12) $$h\left(x\right)=\left[\begin{array}{c} f_{x}x_{1}+c_{u}\\ f_{y}x_{2}+c_{v}\\ 1/x_{3}\\ \psi \\ r_{c} \end{array}\right],$$

where (1), (7), and (9) are used, and $\psi_{m}$ is ψ measured as described in Section 3.2.2, and *d* is the relative distance between the target and the UAV. Measurements *d* and ϕ are defined in detail in Section 3.2.

**Remark** **2.** *Changes in the target velocity can result in inaccurate state estimations, and therefore a UKF [42] is applied to address this issue. The model mismatch between the real and the model defined in (8) is considered as process noise, and the states can be estimated and updated to approach the ground truth in the UKF.*

#### 3.2.2. Orientation Measurement

As shown in Figure 2, $\psi_{m}$ can be obtained from a YOLO network with the captured image from the onboard camera and is used to measure where the UAV is located observed by the camera, so that the controller developed in Section 4 can ensure the control objective $\psi \to \psi_{des}$ defined in (5) can be achieved.

### 3.3. Observability

Since the system dynamics defined in (8) is nonlinear, the approach to evaluate the observability of the system developed in (13) of [43] is applied to establish Theorem 1.

**Theorem** **1.** *The system defined in (8) is observable.
*

**Proof.**  Given an observability matrix *O* defined as

(13) $$O≜\frac{\partial \phi }{\partial x}=\left[\begin{array}{ccc} \frac{\partial L_{f}^{0}\left(h\left(x\right)\right)}{\partial x_{1}} & \cdots & \frac{\partial L_{f}^{0}\left(h\left(x\right)\right)}{\partial x_{n}}\\ \vdots & \ddots & \vdots \\ \frac{\partial L_{f}^{n-1}\left(h\left(x\right)\right)}{\partial x_{1}} & \cdots & \frac{\partial L_{f}^{n-1}\left(h\left(x\right)\right)}{\partial x_{n}} \end{array}\right],$$

where $x_{1},\;\ldots ,\;x_{n}$ are the elements of state x defined in (6), $h\left(x\right)$ is the measurement defined in (12), and $L_{f}^{i}\left(h\left(x\right)\right)$ denoted the *i*th order Lie derivative of $h\left(x\right)$ is defined as

$$\begin{array}{cc} L_{f}^{0}\left(h\left(x\right)\right) & ≜h\left(x\right)\\ L_{f}^{1}\left(h\left(x\right)\right) & ≜\frac{\partial h\left(x\right)}{\partial x}\cdot \;f\\ L_{f}^{2}\left(h\left(x\right)\right) & ≜\frac{\partial }{\partial x}\left[L_{f}^{1}\left(h\left(x\right)\right)\right]\cdot \;f\\ \vdots \\ L_{f}^{k}\left(h\left(x\right)\right) & ≜\frac{\partial }{\partial x}\left[L_{f}^{k-1}\left(h\left(x\right)\right)\right]\cdot f, \end{array}$$

where $f=\dot{x}$ is the vector field, and the submatrix of observability matrix *O* denoted as $O_{s}\in R^{14\times 10}$ is defined as

$$\begin{array}{cc} O_{s} & ≜\left[\begin{array}{c} \frac{\partial L_{f}^{0}\left(h\left(x\right)\right)}{\partial x}\\ \frac{\partial L_{f}^{1}\left(h\left(x\right)\right)}{\partial x} \end{array}\right], \end{array}$$

and can be rewritten as

(14) $$\begin{array}{cc} O_{s} & =\left[\begin{array}{cccccc} f_{x} & 0 & 0 & 0 & 0 & 0\\ 0 & f_{y} & 0 & 0 & 0 & 0\\ 0 & 0 & -x_{3}^{-2} & 0 & 0 & 0\\ \; & \; & & 1 & \; & 0\\ 0 & 0 & 0 & 0 & I_{3} & 0_{3\times 3}\\ - & - & - & - & -\\ \; & \; & \frac{\partial f_{x}{\dot{x}}_{1}}{\partial x}\\ \; & \; & \frac{\partial f_{y}{\dot{x}}_{2}}{\partial x}\\ \; & \; & \frac{\partial \left(-x_{3}^{-2}{\dot{x}}_{3}\right)}{\partial x}\\ \; & \; & \frac{\partial \dot{\psi }}{\partial x}\\ 0 & 0 & 0 & 0 & 0 & I_{3} \end{array}\right]. \end{array}$$

Based on (14), $O_{s}$ is full-rank since rank($O_{s}$) = 10 based on rows 1~7 and rows 12~14, which implies *O* is full-rank matrix and the system is observable.    □

## 4. Controller Architecture

Compared to the existing IBVS control methods [16], the controller developed in this work considers the constraints of visual features and control input in order to improve the tracking performance. The control input is calculated by minimizing the cost function designed in this section that considers not only the error of the feature vector but also the velocity of the moving target. Using the controller can lead to less-aggressive flying behavior and also decrease the energy consumption by choosing appropriate gain matrices in the cost function.

### Controller Design

The dynamics model defined in (8) is derived for the process step in the UKF so that the target state can be estimated. The dynamics model of $s_{m}\left(t\right)={\left[x_{1},\;x_{2},\;x_{3},\;\psi \right]}^{T}\in R^{4}$ used for prediction in the controller can be written as

(15) $${\dot{s}}_{m}=\left[\begin{array}{c} -x_{3}\left(v_{cx}-v_{qx}\right)+x_{1}x_{3}\left(v_{cz}-v_{qz}\right)-\left(1+x_{1}^{2}\right)\omega_{cy}\\ -x_{3}\left(v_{cy}-v_{qy}\right)+x_{2}x_{3}\left(v_{cz}-v_{qz}\right)-\omega_{cy}x_{1}x_{2}\\ x_{3}^{2}\left(v_{cz}-v_{qz}\right)-\omega_{cy}x_{1}x_{3}\\ \frac{\left(v_{cy}^{g}-v_{qy}^{g}\right)\left(x_{c}^{g}-x_{q}^{g}\right)-\left(v_{cx}^{g}-v_{qx}^{g}\right)\left(y_{c}^{g}-y_{q}^{g}\right)}{{\left(x_{c}^{g}-x_{q}^{g}\right)}^{2}+{\left(y_{c}^{g}-y_{q}^{g}\right)}^{2}}-\frac{a_{qy}^{g}v_{qx}^{g}-a_{qx}^{g}v_{qy}^{g}}{{\left(v_{qx}^{g}\right)}^{2}+{\left(v_{qy}^{g}\right)}^{2}} \end{array}\right],$$

where the control input can be reduced to $u≜{\left[v_{qx},\;v_{qy},\;v_{qz},\;\omega_{cy}\right]}^{T}\in R^{4}.$ To deal with the modeling errors associated with the use of the dynamics model and the imperfect actuation of the UAV, an error signal $\epsilon_{k}$ is designed as:

(16) $$\begin{array}{ccc} \epsilon_{k} & = & s_{k}-s_{m,k} \end{array}$$

where $s_{k}$ is estimated using the UKF and $s_{m,k}$ is predicted using the model (15) at time *k*. The desired feature vector utilized in the controller can be defined as

(17) $$\begin{array}{ccc} s_{d,k} & = & s^{*}-\epsilon_{k}, \end{array}$$

where $s^{*}≜{\left[x_{1}^{*},\;x_{2}^{*},\;x_{3}^{*},\;\psi^{*}\right]}^{T}\in R^{4}$ is the reference feature vector prescribed by the user.

With (15) and (17), the optimal control problem (OCP) of the controller can be expressed as

(18) $$\begin{array}{cc} \min_{s_{m},u} & \sum_{i=0}^{N_{p}-1}\left[{∥s_{m,k+i/k}-s_{d,k+i/k}∥}_{Q_{s}}^{2}+{∥u_{k+i/k}-u_{d,k+i/k}∥}_{R_{u}}^{2}\right]\\ \; & +{∥s_{m,k+N_{p}/k}-s_{d,k+N_{p}/k}∥}_{W_{s}}^{2}, \end{array}$$

s.t.

(19) $$\begin{array}{cc} \; & s_{m,k+i+1/k}=F_{m}\left(s_{m,k+i/k},\;u_{k+i/k}\right),\;i=0,\ldots ,N_{p}-1\\ \; & u_{d,k+i+1/k}=u_{d,k+i/k}+{\left[\begin{array}{cc} \ddot{\hat{\Gamma_{i}}}, & 0 \end{array}\right]}^{T}dt,\;i=0,\ldots ,N_{p}-1\\ \; & s_{m,k+i/k}\in S,\;i=1,\ldots ,N_{p}\\ \; & u_{k+i/k}\in U,\;i=0,\ldots ,N_{p}-1\\ \; & s_{d,k+i/k}=s^{*}-\epsilon_{k+i/k},\;i=1,\ldots ,N_{p}\\ \; & s_{m,k/k}=s_{k}\\ \; & s_{d,k/k}=s_{d,k} \end{array}$$

where $u_{d}\in R^{4}$ defined as the desired velocity that control input *u* needs to achieve and can be updated by $\ddot{\hat{\Gamma_{i}}}$ over the prediction horizon, where ${\hat{\Gamma }}_{i}\left(t\right)\in R^{3}$ is an nth-order polynomial obtained by the QP and defined in the interval $\left[t_{i-1},t_{i}\right)$ expressed as

(20) $${\hat{\Gamma }}_{i}\left(t\right)=\sum_{j=0}^{n}c_{ij}{\left(t-t_{i-1}\right)}^{j},\;\mathrm{for}\;t_{i-1}\leq t<t_{i},$$

where $c_{ij}\in R^{3}$ denotes the jth-order coefficient, $S_{m}≜\left\{s_{m,k/k},\;s_{m,k+1/k},\;\ldots ,\;s_{m,k+N_{p}/k}\right\}$ denotes the predicted features at sampling time *k* based on the control sequence $U=\left\{u_{k/k},\;u_{k+1/k},\;\ldots ,\;u_{k+N_{p}-1/k}\right\}$ and the estimation $s_{k}$ obtained using the UKF. $Q_{s}$, $R_{u}$, and $W_{s}$ defined in (18) are positive weighting matrices, and $N_{p}\in N$ is the prediction horizon. Increasing $N_{p}$ results in less-aggressive control inputs but also increases the computational effort. $F_{m}\left(\cdot \right)$ is the model defined in (15). Since only the linear velocity of the target can be estimated (i.e., $\hat{\Gamma_{i}}$), control input $\omega_{cy}$ only depends on the cost of feature errors $s_{m}-s_{d}$. S and U are the sets of constraints of the feature vector and the control input given by

$$\begin{array}{ccc} S & ≜ & \left\{s\in R^{4}|\left[\begin{array}{c} x_{1,min}\\ x_{2,min}\\ x_{3,min}\\ \psi_{min} \end{array}\right]\leq s\leq \left[\begin{array}{c} x_{1,max}\\ x_{2,max}\\ x_{3,max}\\ \psi_{max} \end{array}\right]\right\} \end{array}$$

$$\begin{array}{ccc} U & ≜ & \left\{u\in R^{4}|\left[\begin{array}{c} v_{cx,min}\\ v_{cy,min}\\ v_{cz,min}\\ \omega_{cy,min} \end{array}\right]\leq u\leq \left[\begin{array}{c} v_{cx,max}\\ v_{cy,max}\\ v_{cz,max}\\ \omega_{cy,max} \end{array}\right]\right\}. \end{array}$$

When solving the OCP at time *k*, $\epsilon_{k}$ would be calculated by (16) using the feature vector estimated using the UKF at time *k* and the feature vector predicted by the model (15) at time k−1. This error signal is assumed to be constant over the prediction horizon; $s_{d,k+i/k},\;i=0,\ldots ,N_{p}$ would be constant over the prediction horizon.

The direct multiple shooting method is employed to solve the problem, since “lifting” the OCP to a higher dimension can usually speed up the rate of convergence. The OCP is repeated at every sampling time, with only the first vector $u_{k/k}$ in control set *U* being adopted in the system. Several software libraries can be used to solve this kind of nonlinear programming problem (NLP), and this study used CasADi for formulating the NLP and Ipopt for solving it.

**Figure 4:**
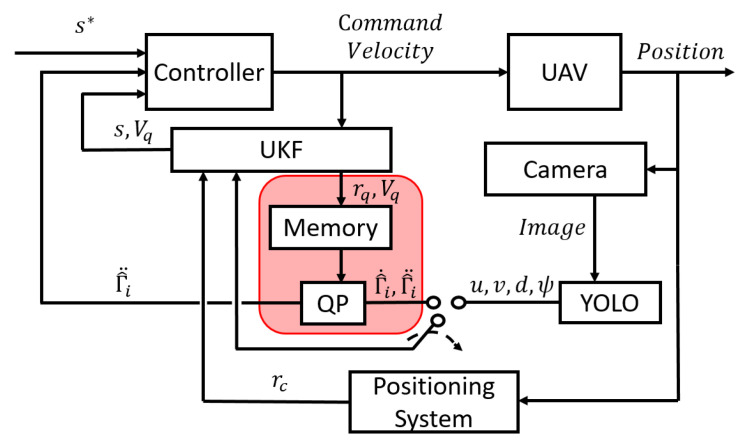
Block diagram of the controller, where u,v,d, and ψ are the measurements being updated using the UKF.

**Remark** **3.** *The control architecture depicted in Figure 4 can be applied to general target tracking. That is, the feature vector and orientation generated from YOLO can be used to predict the motion of target $v_{q},$ which can facilitate tracking performance and, in turn, enhance the detection of bounding boxes.*

## 5. Simulations

### 5.1. Environment Setup

**Figure 5:**
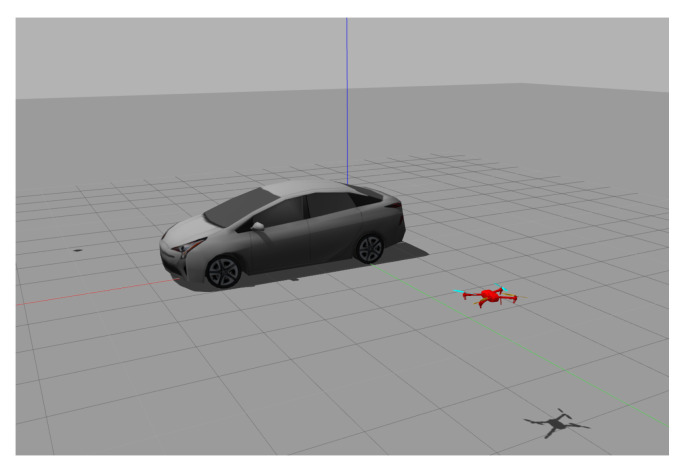
Setup of the car and UAV.

The Gazebo simulator under the ROS framework (version 18.04, Melodic) is used to conduct the simulations to verify the efficacy of the developed control algorithm, and the source code is posted on GitHub for public access (https://github.com/Networked-Control-Robotics-Lab/uav_nmpc_tracking_task.git) (accessed on 5 August 2021). A quadrotor implemented with the developed controller is deployed to track a moving vehicle. The initial locations of the target and the UAV are ${\left[\begin{array}{ccc} 0, & 0, & 0 \end{array}\right]}^{T}$ and ${\left[\begin{array}{ccc} 2.18, & 6.65, & 1.75 \end{array}\right]}^{T}$ and the initial orientations are ${\left[\begin{array}{ccc} 0, & 0, & 90^{\circ } \end{array}\right]}^{T}$ and ${\left[\begin{array}{ccc} 0, & 0, & -90^{\circ } \end{array}\right]}^{T},$ respectively, in the global frame as shown in Figure 5. The target moves on the XY plane following the nonholonomic dynamics, and its velocity is controlled by the user commands with a loop rate of 80 Hz. The resolution of the captured image is 640×480, and the intrinsic parameters of the camera are

$$\begin{array}{ccc} K & = & \left[\begin{array}{ccc} 381.36 & 0 & 320\\ 0 & 381.36 & 240\\ 0 & 0 & 1 \end{array}\right]. \end{array}$$

**Figure 6:**
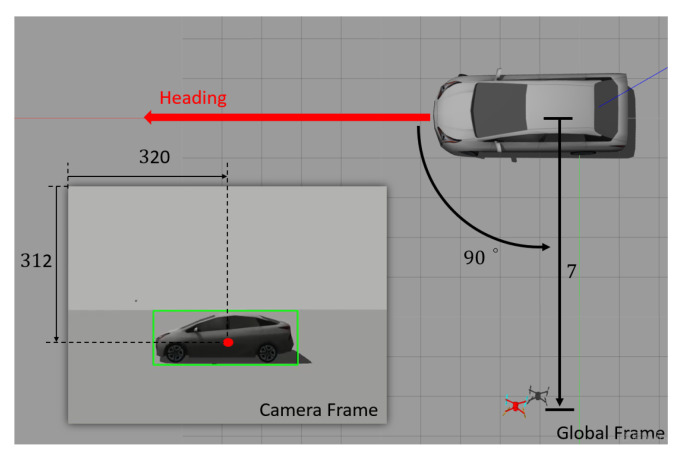
The red dot inside the bounding box represents the reference feature vector as defined in (21) and is used in the simulation as prescribed by the user.

The control goal in the tracking task is to keep the moving target within the FOV of the camera at the desired depth and angle relative to the target. The reference feature vector was

(21) $$s^{*}={\left[\frac{\left(320-320\right)}{381.36},\;\frac{\left(312-240\right)}{381.36},\;1/7,\;90^{\circ }\right]}^{T}\in R^{4}$$

in (17) which defines the desired feature vector, as shown in Figure 6.

In order to maintain the visibility and address the limitations of the actuator while tracking the moving target, the constraints of the states and constraints of the control inputs were considered in the numerical simulations, as listed in Table 1 and Table 2. The parameters for tuning the sampling time, predicted horizons, and the weighting matrices in the cost function are presented in Table 3.

### 5.2. Simulation Results

To demonstrate the practicability and efficiency of the developed controller, three simulations are described that compared the controller and the traditional IBVS controller. In the first simulation, the UAV tracked a target that was at a relative angle that changed over time in order to show the benefit of including the angle dynamics in the dynamics model. In the second simulation, the UAV tracked a target moving over a z-shaped path in order to compare the tracking performance of different designs of controllers in an aggressive-motion condition. In the third simulation, the IBVS controller and the controller were utilized to demonstrate the tracking efficiency in an aggressive-motion condition.

#### 5.2.1. Simulation 1: Controller with Relative Rotational Dynamics

**Figure 7:**
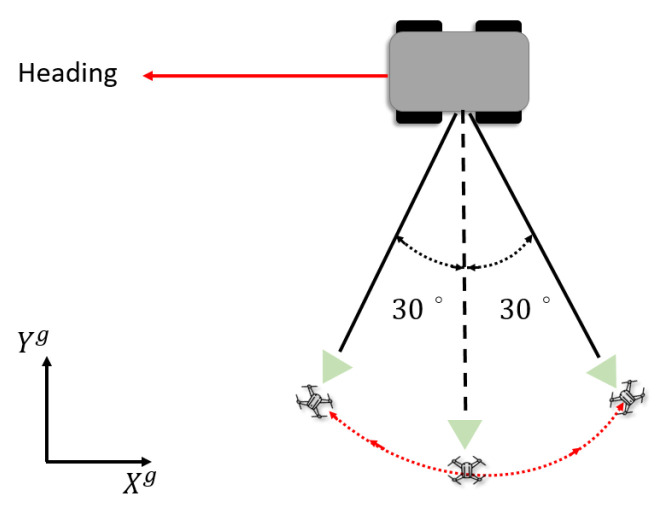
A UAV tracks a target at a desired relative angle that changes over time. The desired relative angle was $\psi^{*}=30\sin\left(t\right)+90$.

The scenario of Simulation 1 is depicted in Figure 7. The controller designed in Section 4 was implemented to track a target at a time-varying angle. In Case 1, the rotational dynamics was not considered in the dynamics model, and the relative orientation was measured directly using a YOLO network. In Case 2, the angle dynamics was taken into consideration in the UKF, which made the relative orientation more robust to noisy or intermittent measurements from the YOLO network.

##### Comparison of State Feature Vectors

**Figure 8:**
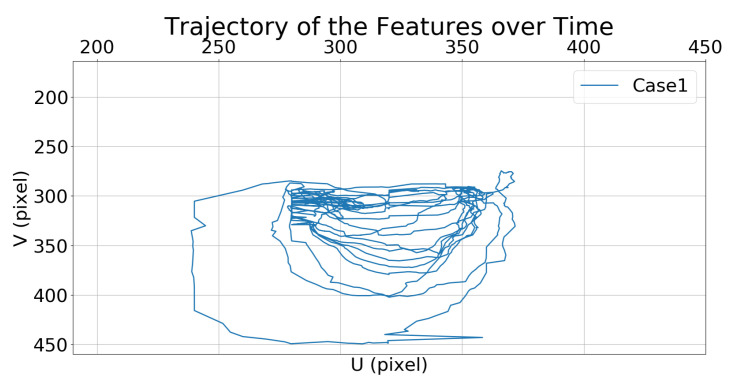
Simulation 1: trajectory of the moving target in the image frame for Case 1.

**Figure 9:**
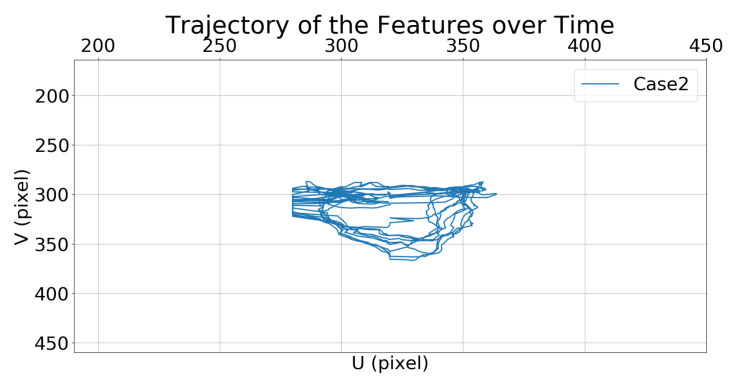
Simulation 1: trajectory of the moving target in the image frame for Case 2.

Figure 8 and Figure 9 show the trajectories of the features (i.e., the center of the bounding box) in the image frame over time.

The performances are quantified as the root mean square (RMS) tracking errors defined in Table 4.

**Figure 10:**
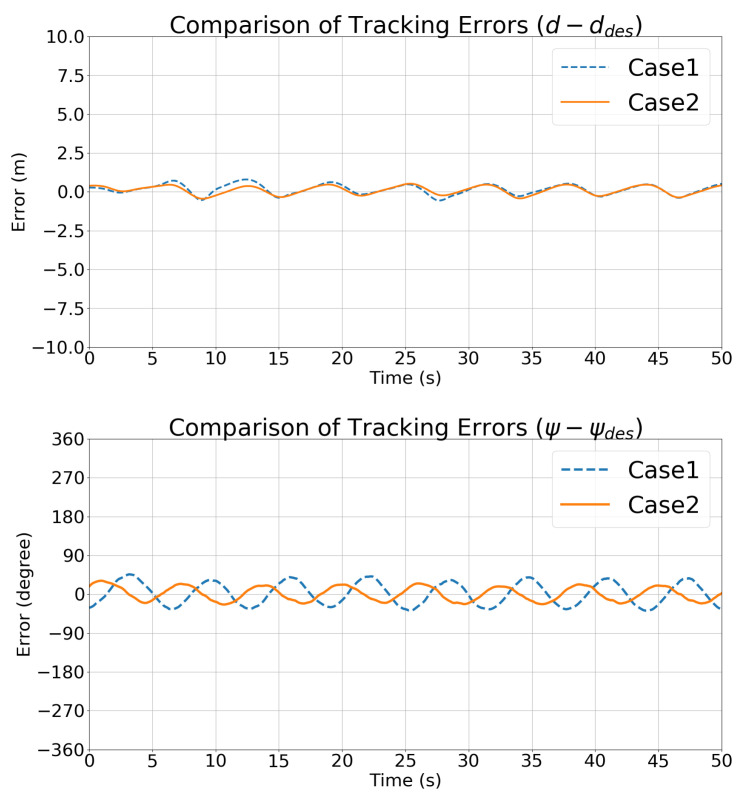
Simulation 1: Comparisons of the state vector tracking errors.

Figure 10 compares the state vector tracking errors in Cases 1 and 2. The trends in the state vectors are consistent in the two cases.

##### Comparison of Control Inputs

**Figure 11:**
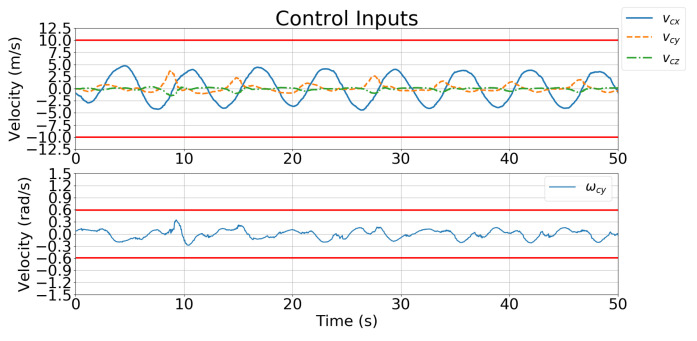
Simulation 1: velocity control input for Case 1.

**Figure 12:**
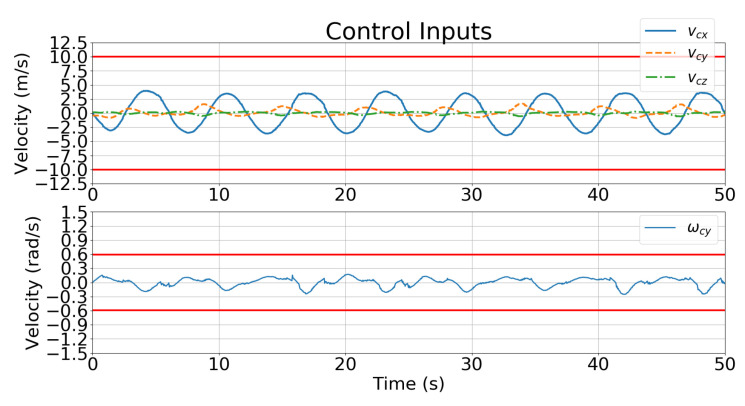
Simulation 1: velocity control input for Case 2.

Figure 11 and Figure 12 show the velocity control inputs for Case 1 and Case 2 in Simulation 1, respectively, and the upper and lower bounds on the control inputs defined in Table 2 are highlighted.

**Figure 13:**
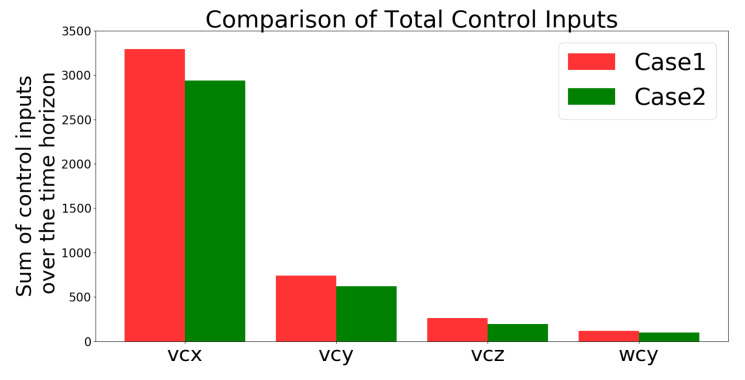
Simulation 1: comparison of the total amount of control inputs.

Figure 13 compares the total control inputs, which shows that they were lower in Case 2 than in Case 1. “The total control inputs are defined as the summation of $u_{k+i/k}$ solved from the cost problem defined in (18) over the simulation time period (i.e., 50 s). The higher the value, the higher the energy consumption since the energy consumption of the UAV is proportional to the cube of its speed $u_{k+i/k}$ defined below (15) based on [44]”.

#### 5.2.2. Simulation 2: Controller with Target Motion Pattern

**Figure 14:**
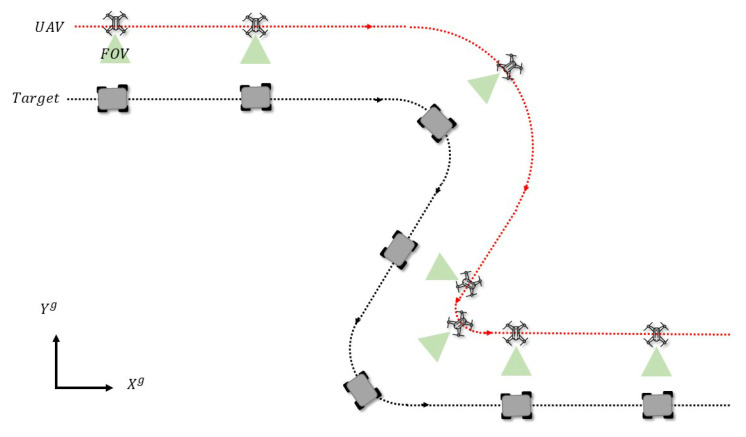
A UAV tracks a moving target moving over a z-shaped path. The trajectory of the target is unknown to the UAV. The target moves at 1 m/s in the heading direction from 3 s to 13 s, and first turns at −0.2 $m/s^{2}$ from 13 s to 23 s. The target keeps moving at 1 m/s from 23 s to 33 s, and performed a second turn at 0.2 $m/s^{2}$ from 33 s to 43 s, and then keeps moving at 1 m/s.

The scenario of Simulation 2 is depicted in Figure 14, where the UAV is controlled to track a target moving over a z-shaped path. The controllers for Cases 1 and 2 are compared. In Case 1, the difference in the contiguous control inputs to the UAV (i.e., differential control cost) is considered in the controller. In Case 2, by leveraging the motion pattern estimated using the UKF, the difference between the velocities of the UAV and the target can be considered in the controller designed in Section 4.

##### Comparison of State Feature Vectors

**Figure 15:**
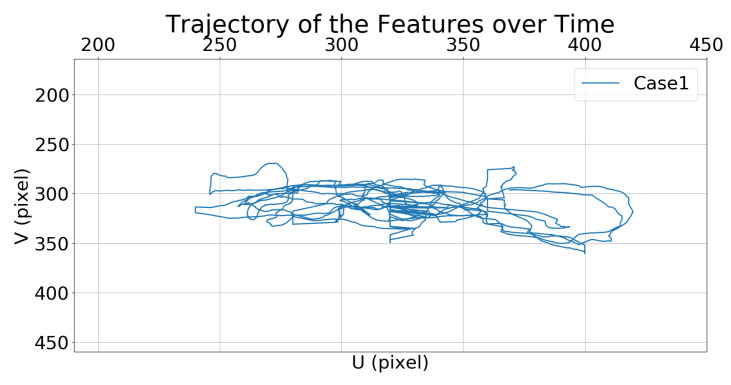
Simulation 2: trajectory of the moving target in the image frame for Case 1.

**Figure 16:**
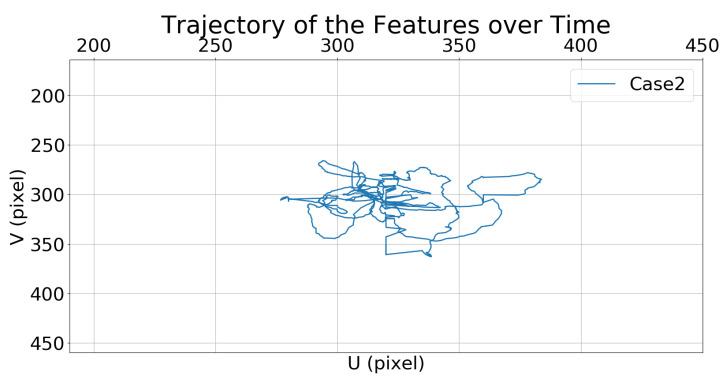
Simulation 2: trajectory of the moving target in the image frame for Case 2.

Figure 15 and Figure 16 show the trajectories of the target features in the image frame over time, which indicate that the feature trajectory in Case 2 moves over a smaller range compared to that in Case 1.

The performances are quantified based on the RMS tracking errors defined in Table 5.

**Figure 17:**
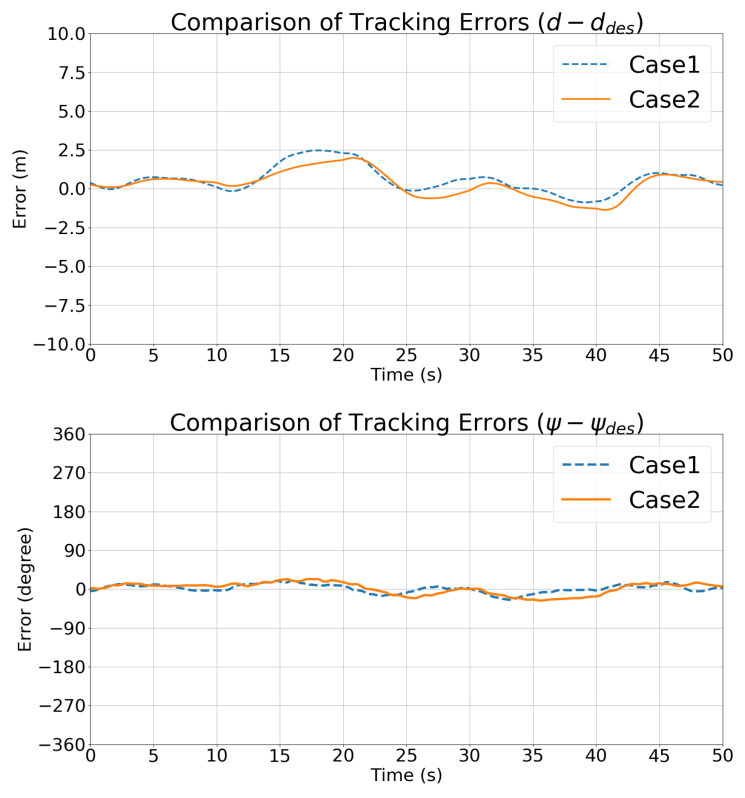
Simulation 2: comparisons of the state vector tracking errors.

Figure 17 compares the depth and relative angle tracking errors in Case 1 and Case 2, which also shows that Case 2 performed better than Case 1. Thanks to the motion pattern estimated using the UKF, the controller developed in Section 4 performed better than the traditional optimal controller with differential control cost.

##### Comparison of Control Inputs

**Figure 18:**
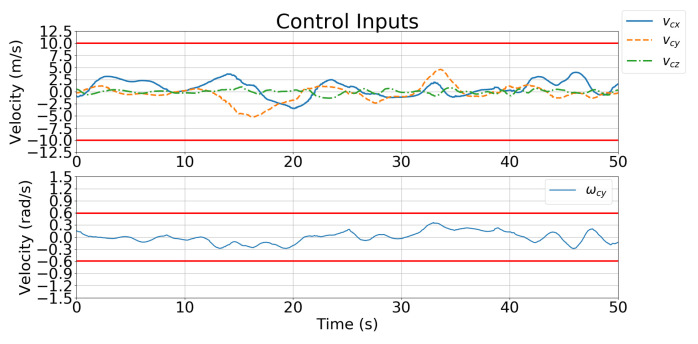
Simulation 2: velocity control input for Case 1.

**Figure 19:**
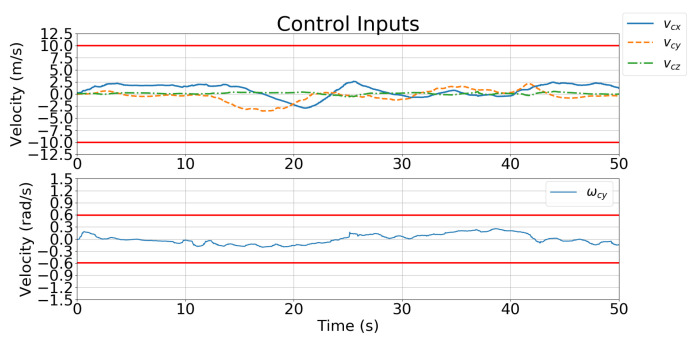
Simulation 2: velocity control input for Case 2.

Figure 18 and Figure 19 show the velocity control inputs for Cases 1 and 2, respectively, and the upper and lower bounds on the control inputs defined in Table 2 are highlighted. The control inputs were much more stable and smoother for Case 2 than for Case 1.

**Figure 20:**
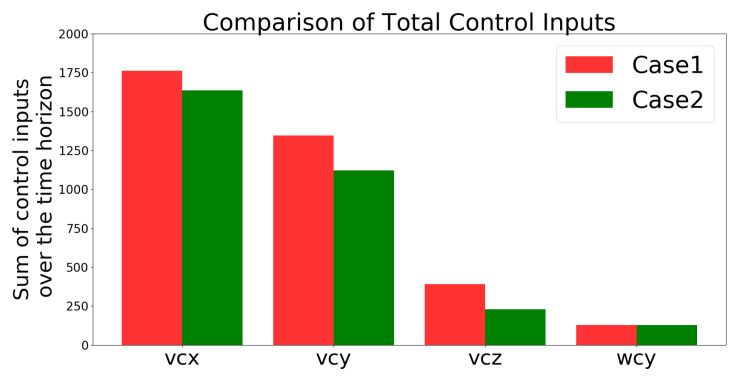
Simulation 2: comparison of the total amount of the control inputs.

Figure 20 compares the total control inputs in Cases 1 and 2, and shows that these inputs were lower for Case 2 than for Case 1.

#### 5.2.3. Simulation 3: IBVS vs. the Developed Controller

The scenario in this simulation is the same as that depicted in Figure 14, but the performances of the IBVS controller and the controller designed in Section 4 are compared to track a target moving over a z-shaped path in Cases 1 and 2.

##### Comparison of State Feature Vectors

**Figure 21:**
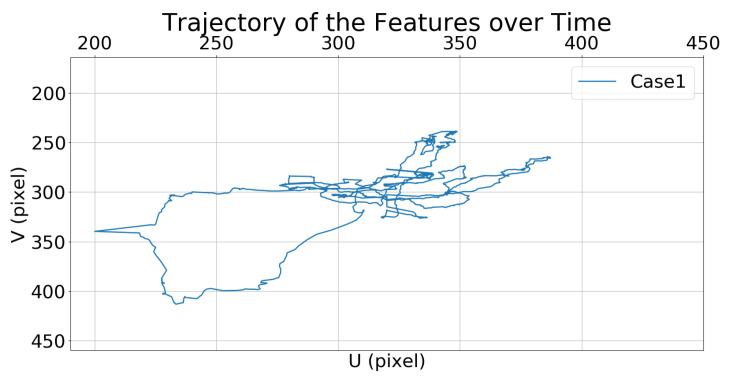
Case 1: trajectory of the moving target in the image frame using the IBVS controller.

**Figure 22:**
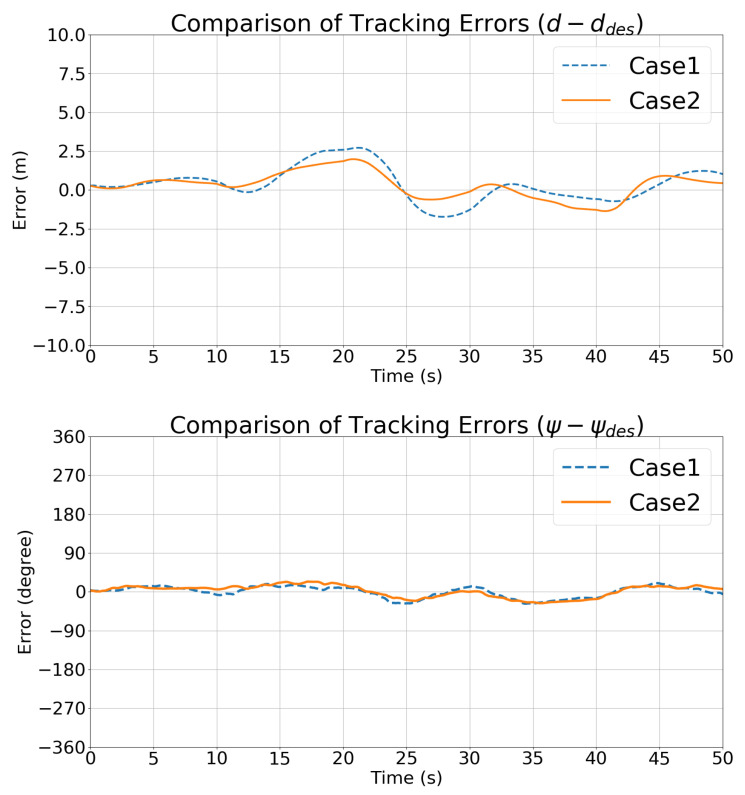
Simulation 3: comparisons of the state vector tracking errors.

Figure 16 and Figure 21 show the trajectories of the features in the image frame over time. The stability of the IBVS controller and the controller is also evident from Table 6, which lists the RMS tracking errors. Figure 22 compares the state vector tracking errors in Cases 1 and 2.

The performances quantified based on the RMS tracking errors are presented in Table 6.

##### Comparison of Control Inputs

**Figure 23:**
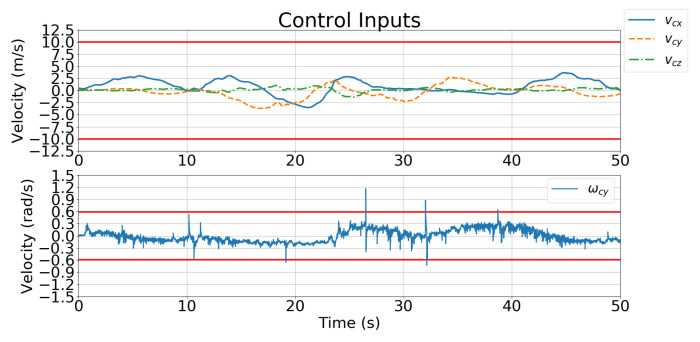
Case 1: velocity control input of the IBVS controller.

Figure 19 and Figure 23 show the velocity control inputs to the IBVS controller and the developed controller, respectively, and the upper and lower bounds on the control inputs defined in Table 2 are highlighted. Figure 23 shows that the linear and angular velocity control input generated for the IBVS controller keeps fluctuating while tracking. In contrast, Figure 19 shows that the linear and angular velocity control inputs of the controller are stable and smooth.

**Figure 24:**
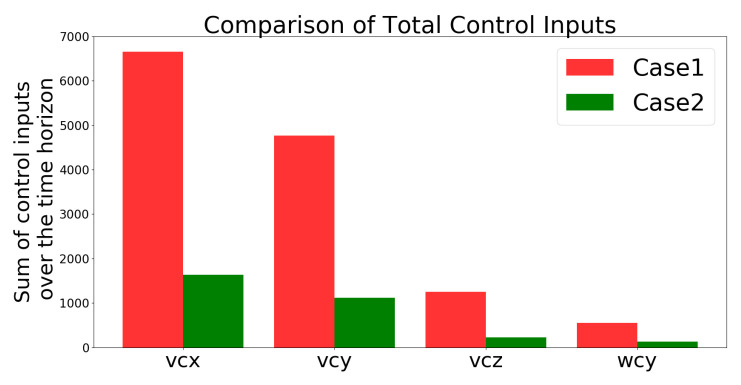
Comparison of the total amounts of the control inputs.

Figure 24 shows that compared with the IBVS controller, the controller needs much less energy to track the moving target. In other words, the controller is much more efficient than the IBVS controller since it saves energy by not generating unnecessary motion.

##### IBVS Controller vs. the Developed Controller

The IBVS controller relies on the image-feature error of the target for feedback, which results in the motion of the UAV becoming unstable and oscillating with the inclusion of a large error (i.e., large displacement and rotation) as shown in Figure 24. In contrast, the developed controller is designed to minimize the cost function that considers not only the current feature error but also predicts the future states of the image features in the predicted horizons according to the dynamics model. Additionally, the constraints of the control inputs are taken into consideration in order to avoid excessive and unreasonable motions. Figure 23 shows that the angular velocity exceeds the limitation of the control inputs defined in Table 2; however, the angular velocity generated by the controller in Figure 19 prevents unreasonable motion from occurring.

## 6. Conclusions

A tracking controller has been developed to track a moving target, and it relies on bounding box features generated by a YOLO network and a target motion pattern obtained by QP, in which the target states are estimated using a UKF. The main features of the developed controller ensure the following characteristics:

1. The control effort is less than for traditional controllers, while the tracking error is quantified by the RMS error and is less than that for a traditional controller.
2. The value of the control input to the controller always remains within the UAV motion capabilities.
3. The target can be tracked, and its image features always remain within the image, which is not guaranteed for a traditional IBVS controller.

All of the above results were verified in three simulations. The first simulation illustrated that the controller, which considers the relative rotational dynamics, can perform better in tracking a target at a specified angle. The second simulation indicated that using the predicted motion pattern of a target in the controller can improve the tracking performance compared to the traditional optimal controller, and the controller also requires less control effort for tracking. The third simulation compared the controller with a traditional IBVS controller, and showed that the controller requires much less control effort and achieves a smaller tracking error. This work has demonstrated both the control efficacy and high performance of the developed controller.

## Figures and Tables

**Table 1:** State Constraints.

States	Min.	Max.
$x_{1}$	−0.84	0.84
$x_{2}$	−0.63	0.63
$x_{3}$	0.07	1
ψ	$0^{\circ }$	$180^{\circ }$

**Table 2:** Control Constraints.

Inputs	Min.	Max.
$v_{cx},v_{cy},v_{cz}$ (m/s)	−10	10
$w_{cy}$ (rad/s)	−0.59	0.59

**Table 3:** Control Parameter Settings.

Parameter	Value
Sampling Time (▵k)	0.025s
Horizon Length ($N_{p}$)	50
$Q_{s}$	$\left[\begin{array}{cccc} 1 & 1 & 1 & 1\\ 1 & 1 & 1 & 1\\ 1 & 1 & 100 & 1\\ 1 & 1 & 1 & 1 \end{array}\right]$
$R_{u}$	$\left[\begin{array}{cccc} 0.02 & 1 & 1 & 1\\ 1 & 0.03 & 1 & 1\\ 1 & 1 & 0.01 & 1\\ 1 & 1 & 1 & 0.3 \end{array}\right]$

**Table 4:** Simulation 1: RMS errors.

Case	*U*-Axis	*V*-Axis
1	29.75	28.93
2	25.00	18.18

**Table 5:** Simulation 2: RMS errors.

Case	*U*-Axis	*V*-Axis
1	40.92	16.60
2	18.80	20.42

**Table 6:** Simulation 3: RMS errors.

Case	*U*-Axis	*V*-Axis
1	34.92	35.23
2	18.80	20.42

## Data Availability

Not applicable.
